# Effects of Instruction‐Guided Attentional Focus on Jump Performance in Women's Artistic Gymnastics

**DOI:** 10.1002/ejsc.70112

**Published:** 2025-12-19

**Authors:** Juliane Veit, Michel Brinkschulte, Tobias Vogt

**Affiliations:** ^1^ Institute of Professional Sport Education and Sport Qualifications, Section Didactics and Methodology in Sports German Sport University Cologne Cologne Germany

**Keywords:** female athletes, movement imagery, stretched jump with turn, vividness

## Abstract

Instructions are a common part of training in many sports. The content of the instructions and how they are formulated is relevant for the effect on performance. For motor learning and skill development, instructions that initiate an external focus of attention are predominantly described as beneficial in the literature. We assume that an instruction‐guided attentional focus causes an effect on jump performance depending on whether the instruction matches the imagery type. Twenty‐nine female participants (*M*_age_ = 19.55 ± 3.01 years) with artistic gymnastics expertise (*M* = 11.38 ± 4.24 years) completed an online questionnaire, the Vividness of Movement Imagery Questionnaire (VMIQ‐2) and performed stretched jumps with a 450° *longitudinal axis* (LA) turn in a laboratory setting. Crucial gymnastic‐specific performance criteria such as jump height, body position and landing details were recorded and analysed. ANOVA results indicate no significant differences in performance between three instruction groups. Results of linear mixed models analyses show differences between baseline performance and the instruction phases for five variables. There seems to be a disruptive effect on performance when attention is consciously directed. The effect of an instruction‐guided attentional focus on performance appears to be independent of the imagery type.

## Highlights

- The baseline testing results show a relationship between the gymnast's expertise and six of the nine performance variables, as well as correlations between imagery vividness and three of the nine performance variables.
- The findings of the intervention suggest a disruptive effect of instruction‐guided attentional focus on jump performance.
- There were no differences in jump performance between the three different instruction groups, regardless of the athletes' imagery type and vividness.

## 1 Introduction

In gymnastics, as in the daily training of many other sports, verbal instructions are used to improve the performance of a skill or movement. The benefit from instructions on learning new skills or improving already learnt skills depends on the type of feedback, amount and content of information, and complexity of the task (Starzak et al. 2022). Previous research suggests that instructional strategies have different effects on novices and experts due to varying levels of automatization, and that the effectiveness of different instructions varies depending on the athletes' level of expertise (e.g., Wulf and Shea 2002; Beilock et al. 2002; R. S. W. Masters 1992). How exactly these instructions are worded is shown to be relevant for the effect on the performance (Werner and Federolf 2023). A verbal instruction can draw athletes' attention to different aspects. The resulting *focus of attention* (FoA) can be drawn either to characteristics of the movement (internal) or to the environment or the movement goal (external). If the internal FoA is used, the athlete pays attention to the movement of their entire body or parts of it. In contrast, the athlete focuses on the environment or result of their movement when they use an external FoA (Chua et al. 2021; Werner and Federolf 2023). As a further option, the FoA can be neutral (Makaruk and Porter 2014).

Many studies suggest that using an external FoA in competition results in better performance compared with an internal or neutral FoA (Chua et al. 2021; Wulf 2013). The external FoA is described as beneficial, regardless of whether motor performance or learning is considered, and also regardless of age, health condition, and level of expertise (Chua et al. 2021). It is assumed that conscious motor control is caused by a movement‐related (internal) FoA that interferes with otherwise automatic processes (an assumption based on the Constrained Action Hypothesis, see Wulf et al. 2001). Therefore, an external FoA could be advantageous for automated movements because it reduces the conscious intervention in the process that controls the movement execution (Wulf et al. 2001; Wulf 2013).

It seems to be common that complex skills require concentration on movement‐relevant aspects or conscious execution for movement form corrections and technique adaptation. Both external and internal focus instruction affect movement form and technique but differed in their impact on it (Werner and Federolf 2023). The effectiveness of a certain FoA depends on both the task requirements and the time in the learning process (Lawrence et al. 2011; Winkelman et al. 2017). Different focus instructions can be used intentionally for different purposes and in different training situations. In an interview with track and field athletes, 84.6% of the participants said that their coaches mainly give body‐related instructions, whereas 69.2% of the athletes specifically stated that they focus internally during the competition (Porter et al. 2010). The question arises if practice contradicts the theoretical models or if coaches and athletes do not exploit the possibilities that science reveals.

Research groups described different impacts of the FoA on motor performance (Bull et al. 2023; Kons et al. 2021), movement kinematics (Lohse et al. 2010) or kinetics (Nadzalan et al. 2020), movement efficiency (Nadzalan et al. 2020), or effectiveness (Nadzalan et al. 2020; Raisbeck and Yamada 2019) and ratings of movement form (Abdollahipour et al. 2015). In the context of artistic gymnastics, some studies with jumping movements can be taken into account. Investigations on jumping skills show better performance achieved with an external FoA (Makaruk et al. 2020; Wulf 2013). In another study on vertical jump performance, the authors concluded that an external FoA showed better performance for the peak power and velocity, whereas an internal FoA showed higher performance for jump height (Kons et al. 2021). Results by Raisbeck and Yamada (2019) suggest that external focus instructions must be specific to the contents of the instruction, to be beneficial for the performance. The landing mechanics of jumps was best when addressed in the instructions, but without reaching the maximum jump height. The improvement in performance is to be expected above all for the aspect addressed in the instruction (Raisbeck and Yamada 2019). The assumption that instructions induce changes precisely in the desired movement part is also supported by Werner and Federolf (2023). They emphasise that it should be taken into account *how* the instruction is formulated exactly as this is of great importance.

However, a challenge stemming from the existing literature is that FoA instructions are not formulated in a standardised way. Simply mentioning body parts in the instruction was shown to be sufficient to allocate an instruction to the internal FoA condition in some studies (e.g., Neumann 2019). In other studies, the internal FoA condition included a specific movement instruction (e.g., Makaruk and Porter 2014). On the other hand, instructions with an external FoA were realised through environmental cues such as ‘focus on jumping close to the cone’ (Marchant et al. 2018) or ‘focus on the direction in which the tape marker is pointing’ (Abdollahipour et al. 2015). Others made reference to the environment without formulating a movement task, such as ‘focus on the movement pathway’ (Lawrence et al. 2011), or with formulating a movement task ‘focus on pushing against the ground’ (Makaruk and Porter 2014). In this regard, Werner and Federolf (2023) state that the different FoA instructions should target the same aspect of movement in the instructional conditions. This indicates that only correctly applied instructions can lead to technical changes that improve athletic performance whereas, unclear, complicated or imprecise instructions can lead to undesirable results (Makaruk and Porter 2014). Such an undesirable effect is also when performance is harmed by instruction‐guided attention. Study results show that an inward directed FoA can deteriorate the performance of experts, by disrupting the automatic skill execution (R. S. W. Masters 1992) whereas novices benefit from skill‐focused instruction (Beilock et al. 2002). During technique training in gymnastics, instruction is used for movement form and technique corrections, partly in a small part of the body or part of the movement. In contrast to the performance outcome, it is not easy to address a technique correction for a certain body part or to target a certain movement part with an externally focused instruction (Neumann 2019). In sports that predominantly require closed motor skills, it could be problematic to draw attention to the movement goal or the movement‐relevant environment. The goal of the task and the way this goal can be achieved are identical for closed motor skills (Hodges and Franks 2002). For artistic gymnastics—assuming it predominantly involves closed skills—it can therefore be concluded that an internal imagery use is most expedient.

For learning novel gymnastics routines, Lawrence et al. (2011) observed that a task‐irrelevant internal focus can lead to a significant improvement in the movement, whereas an external FoA can worsen performance. In their study, gymnasts performed a routine that was executed with an internal skill‐relevant, internal skill‐irrelevant, external, or no FoA. In the repetition and transfer test, the results showed no significant differences. Other results have been observed with 12 year old experienced gymnasts performing a maximum vertical jump with a 180° longitudinal axis (LA) turn (Abdollahipour et al. 2015). Participants performed trials under external and internal FoA and control conditions. Best results in jump height and movement form were reported for the external focus condition. However, a review by Werner and Federolf (2023) revealed that internal focused instructions seem to be more applicable to direct movement form changes than external focused, stating that a general superiority of [external focus] instructions in technique training is not sufficiently legitimate” (p. 8). Perhaps, other aspects need to be considered here because in artistic gymnastics, movement form corrections and technique adaptation is an important aim of instructions.

To investigate the different FoA subtypes, imagery is suggested as a possible approach (Gose and Abraham 2021). In their work, Makaruk and Porter (2014) discuss if a certain FoA is more target orientated with an adapted movement imagery type. A few studies have already investigated whether internal or external FoA influences the performance dependent on their imagery modality dominance. It was shown for throwing accuracy that a higher level of external‐visual imagery dominance resulted in greater motor learning for children adopting an external FoA. Children with higher values of kinaesthetic imagery dominance who adopted an external FoA showed reduced motor learning performance (Bahmani et al. 2021). A change in imagery perspective preference with a trend from a more internal to a more external imagery perspective with ageing (Liu et al. 2019) and a transfer from a visual to a kinaesthetic imagery was observed in older subjects compared with younger ones (Subirats et al. 2018). Results from a study of gymnasts (Veit and Vogt 2025) show that no imagery type is rated as more vivid based on age or expertise, but participants reported different frequencies of imagery use in training. However, the gymnasts did rate the imagery types differently depending on whether they had experience as a coach. Gymnasts who were simultaneously active athletes and coaches rated kinaesthetic imagery as less vivid compared with gymnasts without current coaching responsibilities (Veit and Vogt 2025). A possible explanation is that coaching requires observing and analysing movements from an external perspective, which may reduce the reliance on other imagery types.

Coaches often advise athletes to visualise a skill before executing it in training. However, it is not always considered where exactly to focus one's attention while visualising. Therefore, the question arises whether a specific FoA when visualising can improve the quality of the skill in different ways. Does performance deteriorate when a gymnast is instructed to direct their FoA inward, even though their external imagery is most vivid, and is it really necessary for improved performance that the instruction‐guided FoA matches the individual type of mental imagery? To answer this question, the present study aims to investigate whether a specific imagery type is useful for the instruction‐guided attentional focus. Based on the differing results in the literature for various sport‐specific situations, we examine the stretch jump in gymnastics. Findings in this regard can help coaches to formulate instructions in practice more individualised and effective for skill development. In addition, we assess if better gymnastic jump performance is achieved with more vivid imagery or which variables determine gymnastic jump performance. We assume that a gymnast's age, expertise and imagery vividness affects performance.

Due to the large number of studies with jump investigations and the sport‐specific characteristics of the skill, we chose a stretch jump movement with 450° LA turn for the present study. A stretch jump with 360° LA turn is classified as a simple element (i.e., A) according to the Women's Artistic Gymnastics Competition Rules Code of Points by the Fédération International de Gymnastique (Fédération International de Gymnastique 2020). Although the stretch jump with LA turn is a basic jump, it is shown at different levels, in different variations of execution (i.e., with split during jump), and both on floor and balance beam. It includes typical gymnastic features such as a flight phase and a rotation. At the national level, there are also more simple elements, such as a stretch jump with a smaller or without a LA turn. Regular stretch jumps in women's artistic gymnastics are usually exercised with a 360° or 540° LA turn. In a 450° LA turn, a fully automated movement execution should therefore be prevented. Taking the Constrained Action Hypothesis (Wulf et al. 2001) into account, the question arises as to whether the movement is automated to such an extent that it benefits from an external focus or whether conscious control by an internal focus is advantageous. Based on the theoretical considerations, we formulated the following hypothesis:

Different instruction‐guided FoA (external, internal, neutral) lead to differences in performances when executing stretch jumps with a 450° LA turn, depending on which imagery type (external, internal, kinaesthetic) is most vivid.

## 2 Materials and Methods

### 2.1 Participant Selection

Twenty‐nine female athletes between 14 and 24 years old (19.55 ± 3.01), with women's artistic gymnastics expertise of at least 5 years (11.38 ± 4.24) participated in the study. Participation was limited to female skilled athletes, because their performance would not be comparable to that of male artistic gymnastics athletes, due to different execution and judging criteria. All athletes competed at least in regional competitions but on different levels and were from different German gymnastics clubs. The age range 14–24 was a including criterion and was specified for covering different age groups (in artistic gymnastics, athletes are divided into age groups for competitions). Another criterion for participation was that the athletes must currently be able to perform the movement under investigation, and have been able to do so for at least 1 year, to ensure the most stable technical execution possible.

### 2.2 Procedure and Data Collection

Our study consists of an online questionnaire and a practical part (called *450° Jump Test* in the subsequent parts of the manuscript) with baseline testing and intervention. Participants were informed about the procedures and tasks before starting the experiment. Participants agreed to take part voluntarily and were informed that they could withdraw at any time without providing a reason. This research was conducted in accordance with the Declaration of Helsinki and was approved by the ethics committee of our university. The following describes the procedure of both parts.

#### 2.2.1 Online Questionnaire

In the first part of the study, we conducted an online survey was conducted to collect sociodemographic data, and sports‐specific information, followed by questions on movement imagery vividness. For our investigation, we used the German version of the Vividness of Movement Imagery Questionnaire (VMIQ‐2; Dahm et al. 2019; see Roberts et al. 2008 for the original English version), to assess the ability to visualise a variety of movements. The VMIQ‐2 assesses the ability to form mental images of 12 motor tasks (i.e., running, kicking a stone, jumping sideways) for three imagery types: external–visual imagery (EVI), internal–visual imagery (IVI), and kinaesthetic imagery (KI). For this study, one item with the analysed gymnastic‐specific skill (i.e., stretched jumps with LA turn) was added. Participants were asked to imagine 13 different motor tasks in each of the three imagery types (EVI, IVI, or KI). For each motor task, they rated the vividness of movement imagery on a five‐point Likert scale, (1 = *perfectly clear and vivid*; 5 = *no image at all, I only know that I am thinking about the movement*).

For the intervention athletes were subdivided into four imagery groups, based on the most vivid rated imagery type in the VMIQ‐2 (EVI, IVI, or KI), or if the athletes rated two or three imagery types equally, they were assigned to a ‘Mix’ group.

#### 2.2.2 450° Jump Test

In the intervention, athletes performed a stretch jump with a 450° LA turn. All trials were recorded with two cameras. Camera 1 (Apple iPadPro 12.9″, resolution 1080p, 60fps), placed at a distance of 3 m and height of 1.5 m, captured the entire body from a frontal perspective at the start position, to analyse the body position during the jump. Camera 2 (Apple iPadAir2 9.7″, resolution 1080p, 60fps) placed at a distance of 1.5 m captured the feet from a frontal perspective at landing position.

All trials were analysed for three sport‐specific criteria, based on the criteria of the current FIG code of points: jump height, body position, and landing of the jump. According to the Code of Points, the deviation from the LA turn is the recognition criterion for this skill (Fédération International de Gymnastique 2020). The most important details during the jump are an upright body, hip extension, closed legs, and straight knees. The required execution for landing were closed legs and landing without further adjustment of the feet (Fédération International de Gymnastique 2020).

The jumps were performed between two optojump measuring devices (Microgate, Italy) to determine the jump height. The system measures the flight time and calculates the jump height in centimetres. Camera 1 was used to analyse the body position during the jump, and camera 2 the landing. For the analysis, a video computerized method was used, the tool Kinovea (2021), to observe the technique. The performance variables were measures on the computer using the video analysis software‐supported tool and are described in Table 1. There were predominant joint angles that were measured by placing marks by a gymnastics expert (national licence for judging and coaching in women's artistic gymnastics).

**TABLE 1:** Description, abbreviation, explanation, and measurement of the variables.

Variable	Abb.	Explanation	Measured in
Jump height	Height	Measured with optojump devices	centimetres
Longitudinal axis	LAD	Deviation from aimed 450° LA turn, (determined for front‐foot in direction of movement‐ from take‐off to landing)	degrees
Hip angle	Hip	Determined after 270° of rotation by the angle between the upper body and thigh, 180° is optimum	degrees
Body alignment 270°	Body1	Forward (+) or reverse (−) deviation from the vertical, determined after 270° of rotation, 0° is optimum	degrees
Body alignment 360°	Body2	Sideways deviation from the vertical in (+) or against (−) the direction of rotation, determined after 360° of rotation, 0° is optimum	degrees
Knee angle	Knee	Determined for the more bent one, 180° is optimum	degrees
Leg/knee separation	Legs1	No separation versus less than shoulder width	—
Closed legs at landing	Legs2	Closed versus apart at first contact of landing	—
Steps after landing	Steps	Extra steps after landing	Number

The participants started with a baseline test. After a warm up and one test trial, the athletes were asked to perform six stretched jumps with a 450° LA turn. Between each trial, they took a ten‐second break to recover and focus on the next jump. No advice or feedback was given during the trials.

Following the baseline test, the intervention was carried out, in which the athletes performed four rounds of five jumps. Before each round, the athletes received an instruction presented on a screen. Between each trial, they took a ten‐second break. No augmented feedback was given. A manipulation check (as in Lawrence et al. 2011) was carried out to state the intensity of their FoA after each block, with a five‐point Likert‐scale. On a scale from 0 to 4, participants were asked to indicate the extent to which they were able to adopt the appropriate FoA. Afterwards, the next round started, always with reading the instruction. The instruction remained the same over the four rounds. There were three different instructions, each for a different group, aimed at initiating a different FoA but in line with the same movement goal, the realisation of the LA turn. The instruction formulations were derived from Chua et al. (2021), who differentiate between an internal and external FoA in such a way that the focus is either on movements of body parts or on intended movement effects. The instructions were formulated according to the two investigated visual imagery perspectives (external and internal focussed instruction), complemented by a neutral instruction.

Instructions were given as follows (English translation provided for the purpose of comprehensibility, German original used in the experiment in square brackets):

Perform the stretch jump with 1 ¼ turn and focus your attention on …

[Turne den Strecksprung mit 1¼ Drehung und richte deine Aufmerksamkeit auf…]

- ‘… the path that your shoulders move along around your body’ (external instruction)
  [‘… den Weg den deine Schultern um dich herumziehen’]
- ‘… the rotation movement of your upper body’ (internal instruction)
  [‘… die Drehbewegung deines Oberkörpers’]
- ‘… the movement’ (neutral instruction)
  [‘… die Bewegung’]

**FIGURE 1:**
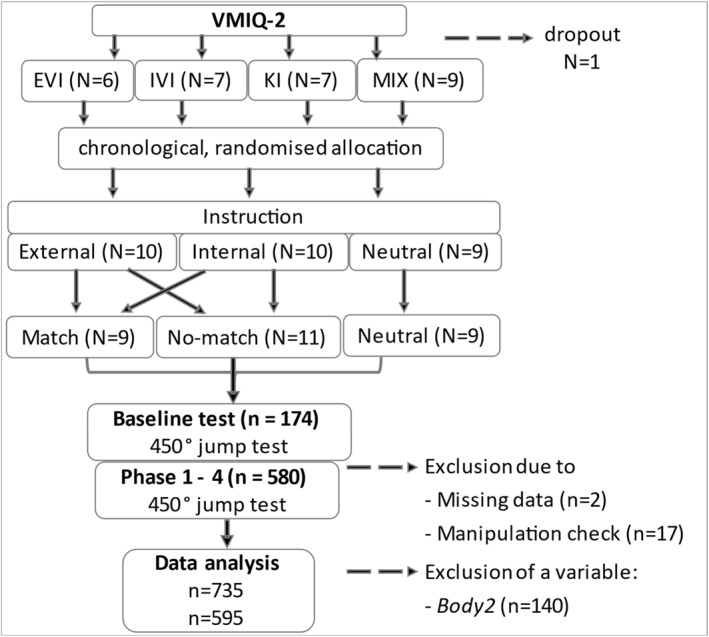
Flowchart illustrating distribution of imagery types (EVI, external–visual imagery, IVI, internal–visual imagery, KI, kinaesthetic imagery, MIX, two or three imagery types equally) to instructions groups and data collection process. *N* indicates the size of the subsample, and *n* the number of trials. VMIQ‐2: Vividness of movement imagery questionnaire.

The three different instructions were systematically distributed over the imagery groups, so that participants with each most vivid imagery type were evenly assigned to all instruction types (see Figure 1). For example, participants with most vivid external imagery were evenly distributed across the external, internal, and neutral instruction group. The same procedure was followed for all of the other imagery groups, including the mixed group. The plan was to cover each instruction with 10 athletes, but in the neutral case there were only 9. All participants in the mixed imagery group were considered to the match group if they received external or internal instructions and if the corresponding imagery was also one of the most vivid imageries. This distribution results in three instruction groups for the analysis (see Table 2). A *match group* (instruction‐guided attentional focus that fits the most vivid imagery type), a *no‐match group* (the instruction‐guided attentional focus is contrary to most vivid imagery type), and a *neutral group* (neutral instruction). Consequently, all participants with kinaesthetic imagery were categorised to the no‐match group, as there was no matching instruction.

**TABLE 2:** Descriptive statistics for age, expertise, and vividness as a function of instruction group.

Participant information	All (*N* = 29)	Match (*N* = 9)	No‐match (*N* = 11)	Neutral (*N* = 9)
	*M* (SD)	*M* (SD)	*M* (SD)	*M* (SD)
Age (years)	19.55 (3.01)	20.22 (3.73)	18.64 (3.11)	20.00 (1.94)
Expertise (years)	11.38 (4.24)	13.11 (5.93)	11.18 (2.80)	9.89 (3.48)
Vividness (five‐point scale)	1.55 (0.60)	1.26 (0.30)	1.50 (0.23)	1.91 (0.93)

*Note: N* indicates the size of the (sub)sample.

### 2.3 Data Analysis

When preparing the data set for analysis, individual jumps were excluded for the final analysis if (1) the software did not record parts of the data, (2) the jump could not be performed in line with the criteria (e.g., the LA turn was not performed wide enough for the measuring system to correctly determine the body position), or (3) the desired instruction‐guided attentional focus could not be ensured (athletes ticked the value ‘0’ in the manipulation check).

We included the data from all 29 participants in the data analyses, resulting in a total of 754 performed jumps. We had to exclude 19 observations due to technical issues (2 trials from two participants) or the manipulation check (17 trials from three participants, two from no‐match group, one from neutral group, in Phase 5 and partly Phase 4), leaving 735 observations for data analysis. For the performance variable Body2, we needed to exclude 140 data points (from 17 participants, most from match group (48) and no‐match group (67), most trials in Phase 3, 4 and 5) because the deviation from the targeted LA turn was higher than −90°, which made the values too large to analyse (i.e., the camera position did not allow for a correct analysis). As we did not find any systematic patterns for these missing data points, we did not exclude the entire observation and therefore handled it as missing completely at random. In this case, the sample size allowed for carrying out the corresponding analyses related to Body2 with all remaining 595 observation.

Collected data were analysed using the IBM SPSS statistics (IBM Corp 2023). Descriptive data in figures and tables are presented as mean (*M*) with standard deviation (SD). In line with statistical convention the level of significance was set at *α* = 0.05 for inferential analyses. Statistical assumptions for conducting analyses of variance (ANOVA) and linear mixed effects models were checked and found to be met for all carried out analyses.

First, one‐way ANOVAs were completed to test for group differences that could potentially confound the main results. Here, we tested for differences within the *imagery* and *instruction groups* regarding the dependent variables *age*, *expertise,* and *imagery vividness*. Then, we used Pearson (for the metric performance variables) and Spearman (for the nominal performance variables) correlations to determine possible correlations between gymnast's characteristics (*age, expertise,* and *vividness of imagery*) and performance at baseline testing to rule out further possible confounds. Statistical effects were analysed using a linear mixed‐effects modelling approach. Participants were modelled as random effects to account for repeated measures effects whereas *instruction group* and *phase* were modelled as fixed effects. In order to examine the relationship between the instruction groups and the performance, linear mixed effects models were fit for the nine different dependent variables. Effect sizes are presented and interpreted in line with statistical convention, based on Cohen (1988).

## 3 Results

Descriptive data for *age*, *expertise,* and *imagery vividness* as a function of *instruction group* are presented in Table 2.

ANOVA results revealed that the instruction groups do not differ at *age* (F(2, 26) = 0.823, *p* = 0.450, *η* ^2^ = 0.060), *expertise* (F(2, 26) = 1.352, *p* = 0.276, *η* ^2^ = 0.094), or *imagery vividness* (F(2, 26) = 3.075, *p* = 0.063, *η* ^2^ = 0.191). Also ANOVA results revealed that the four imagery type groups did not differ at *age* (F(3, 25) = 0.256, *p* = 0.856, *η* ^2^ = 0.030), *expertise* (F(3, 25) = 0.242, *p* = 0.866, *η* ^2^ = 0.028), or *imagery vividness* (F(3, 25) = 0.010, *p* = 0.999, *η* ^2^ = 0.001). At the descriptive level, the average values of the manipulation checks decreased over the four rounds across all participants (*M* = 2.62 ± 0.82 in round one; *M* = 2.35 ± 1.00 in round two; *M* = 2.02 ± 1.02 in round three; *M* = 1.91 ± 1.15 in round four). The manipulation check scores were used as exclusion criteria (by score ‘0’) but were not considered in the statistical analysis.

### 3.1 Performance Data

**FIGURE 2:**
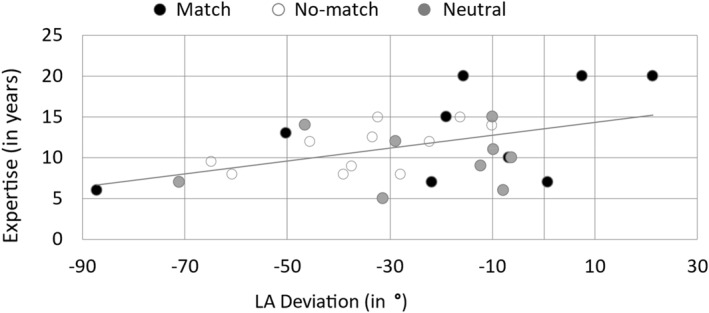
Correlation between expertise with deviation from 450° LA turn across instruction groups’ match, no‐match, and neutral.

The results show that the level of *expertise* is significantly correlated with six performance variables at baseline testing: *LAD* (*r* = 0.374, *p* < 0.001, *n* = 174), *Hip* (*r* = 0.399, *p* < 0.001, *n* = 174), *Knee* (*r* = 0.374, *p* < 0.001, *n* = 174), *Body1* (*r* = 0.278, *p* < 0.001, *n* = 174), *Legs1* (*r* = 0.158, *p* = 0.038, *n* = 174), and *Steps* (*r* = −0.238, *p* = 0.002, *n* = 174). The variable *age* is significantly correlated with *Height* (*r* = 0.199, *p* = 0.009, *n* = 173) and *Knee* (*r* = 0.412, *p* < 0.001, *n* = 174). Figure 2 shows the relationship between gymnast's *expertise* and our main dependent variable *LAD*. Pearson correlations show significant results for *imagery vividness* and three performance variables at baseline testing: *Height* (*r* = 0.156, *p* = 0.040, *n* = 173), *Body1* (*r* = −0.267, *p* < 0.001, *n* = 174), *Body2* (*r* = −0.151, *p* = 0.48, *n* = 174).

Descriptive statistics of the performance variables for all participants and trials are shown in Table 3, separately for baseline testing and phases 1–4. This table therefore shows the mean values for all dependent variables when considering the entire sample.

**TABLE 3:** Descriptive statistics of the nine performance variables as a function of phase.

			Base	1	2	3	4	All
LAD	(In °)	*M* (SD)	−27.10 (29.16)	−36.14 (39.49)	−49.36 (45.50)	−61.05 (42.48)	−65.15 (36.12)	−46.55 (41.25)
Height	(In cm)	*M* (SD)	22.73 (3.27)	22.25 (3.41)	21.99 (3.76)	21.56 (3.68)	21.40 (3.55)	22.03 (3.55)
Hip	(In °)	*M* (SD)	172.95 (5.03)	172.46 (4.99)	171.15 (6.79)	170.92 (7.32)	171.27 (5.84)	171.81 (6.07)
Knee	(In °)	*M* (SD)	171.69 (7.38)	172.05 (5.10)	171.29 (5.98)	170.76 (6.27)	171.65 (5.68)	171.49 (6.19)
Body1	(In °)	*M* (SD)	3.26 (2.23)	3.80 (3.28)	4.35 (3.59)	5.22 (4.48)	6.14 (5.04)	4.47 (3.89)
Body2	(In °)	*M* (SD)	3.70 (2.62)	4.65 (3.46)	4.72 (3.96)	5.68 (4.65)	5.86 (4.80)	4.73 (3.86)
Steps	(Number)	*M* (SD)	0.65 (0.56)	0.93 (0.83)	1.37 (1.61)	1.28 (0.79)	1.33 (0.94)	1.09 (0.25)
Legs1	(In %)		58	58	52	50	53	54
Legs2	(In %)		17	3	4	3	4	7

**FIGURE 3:**
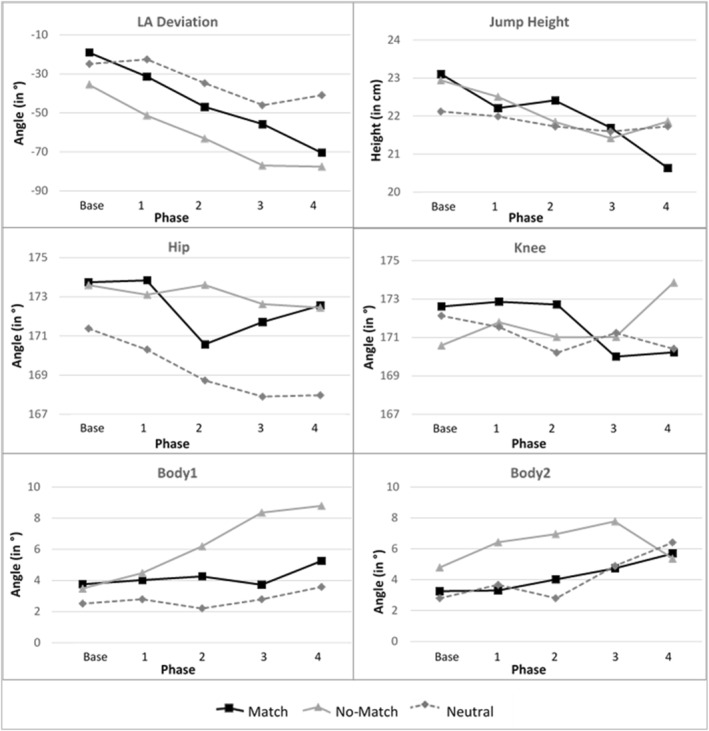
Comparison of the descriptive statistics for the tested performance variables between the three instruction groups across base line testing and phases 1–4.

For a more detailed visualisation of the performance, Figure 3 shows the development over the course of the phases (baseline testing and phases 1–4) for the six metric variables comparing between the three instruction groups.

Linear mixed model results show no significant differences between the three *instruction groups* in the metric performance *variables LAD* (χ^2^(2) = 1.735, *p* = 0.200), *Height* (χ^2^(2) = 0.012, *p* = 0.913), *Hip* (χ^2^(2) = 0.001, *p* = 0.974), *Knee* (χ^2^(2) = 0.061, *p* = 0.806), *Body1* (χ^2^(2) = 1.570, *p* = 0.222), and *Body2* (χ^2^(2) = 0.755, *p* = 0.394). Logistic regression model results show no significant differences for the nominal performance variables *Legs1* (χ^2^(2) = 0.260, *p* = 0.771), *Legs2* (χ^2^(2) = 1.175, *p* = 0.309), and *Steps* (χ^2^(2) = 1.093, *p* = 0.336).

Linear mixed‐model results reveal significant performance differences between the *phases* for the five variables *LAD* (χ^2^(4) = 75.079, *p* < 0.001), *Height* (χ^2^(4) = 19.104, *p* < 0.001), *Hip* (χ^2^(4) = 6.448, *p* < 0.001), *Body1* (χ^2^(4) = 14.756, *p* < 0.001), and *Body2* (χ^2^(4) = 6.667, *p* < 0.001), but no significant performance variable *Knee* (χ^2^(4) = 2.058, *p* = 0.085). The parameter estimates for the linear mixed models are displayed in Table 4. The detailed mixed model results are available from the corresponding author upon reasonable request.

**TABLE 4:** Parameter estimates and *p*‐values of predictors for linear mixed models for significant metric variables.

	Fixed effects	Constant	Base	Phase 1	Phase 2	Phase 3	Phase 4
LAD	Estim.	−55.50	40.29	31.07	18.03	5.66	0^a^
	*p*	< 0.001*	< 0.001*	< 0.001*	< 0.001*	0.051	
Height	Estim.	20.95	1.57	1.05	0.82	0.37	0^a^
	*p*	< 0.001*	< 0.001*	< 0.001*	< 0.001*	0.074	
Hip	Estim.	139.15	2.36	14.18	12.79	0.18	0^a^
	*p*	< 0.001*	0.539	< 0.001*	0.001*	0.963	
Knee	Estim.	170.79	0.537	0.829	0.136	−0.453	0^a^
	*p*	< 0.001*	0.271	0.103	0.788	0.373	
Body1	Estim.	1.45	−3.05	−1.50	−1.02	−0.26	0^a^
	*p*	0.322	< 0.001*	0.002*	0.034*	0.577	
Body2	Estim.	−4.54	2.17	0.94	0.58	0.20	0^a^
	*p*	0.008*	< 0.001*	0.075	0.298	0.712	

*Note:* The letter ^a^ indicates that this parameter is set to zero because it is redundant.

## 4 Discussion

The aim of this study was to investigate which instruction‐guided attentional focus causes an effect on jump performance and to ascertain whether an instruction matched with the most vivid imagery type results in better jump performance than one that either does not fit or is a neutral instruction. Findings in this regard can help coaches to formulate more individualised and effective instructions for skill development, for example, by specifically adapting the instruction‐guided attentional focus to the individual imagery types. Contrary to the hypothesis, our results do not indicate a superiority of an instruction condition, neither with nor without reference to imagery type or vividness. The instruction groups did not differ in age, expertise, or imagery vividness, which indicates that the groups did not differ in their participant characteristics. Possible effects due to an unequal distribution of participants in the subsamples can therefore be ruled out.

The results reveal a significant decline in performance over the course of the phases. Overall, the deterioration was observed regardless of the instruction and the FoA condition. In five of the nine dependent variables, the performance deteriorated significantly from the baseline test over the four phases. The decreasing performance implies fatigue caused by the number of repetitions in the experimental setup, an effect that is well‐known from different contexts (e.g., Aune et al. 2008; Berger and Smith‐Hale 1991; Lorist et al. 2002). However, our data do not allow for an insight of whether muscular fatigue, loss of concentration, or any other disturbance caused the decline movement execution. The exercise science literature discusses three main types of fatigue in this regard, namely physical, mental, and neuromuscular fatigue. Studies have shown that mental fatigue can negatively impact various aspects of physical performance (Zardosht et al. 2025). Other observations suggest that mental fatigue impairs sport‐related performance during exercises performed at a submaximal intensity (Pageaux and Lepers 2018). This could also apply to our study in which participants perform a considerable number of repetitions at submaximal intensity. It is possible that the effects of the instruction are outweighed by fatigue, leading to null findings. Further biomechanical changes are associated with changes in neuromuscular control strategies that can occur during sport‐specific tasks (Fort‐Vanmeerhaeghe et al. 2023). To a certain degree, changes in body angles (e.g., in the variables *Hip* and *Body1*) were observed in the present study. Another line of research has indicated that motor imagery can result in the inhibition of motor commands (see Scheil and Liefooghe 2018), which could be a potential explanation for the performance decline in our study. For fatigue reduction, a systematic review suggests a cluster set design as a preventive measure to reduce the decline in jump performance (Marshall et al. 2021). However, the results of our study imply fatigue effects even though we used such a cluster set design.

Further, the results show that *age*, *level of expertise,* and *imagery vividness* is significantly correlated with various performance variables. This means that all athletes were all able to perform the movement, even if they were unfamiliar with the exact execution of this particular jump (gymnasts usually perform 360° or 540° LA turns), but their performance differed depending on their socio‐demographic characteristics. The task was to execute the stretch jump with LA turn as accurately as possible whereas body posture and the ability to stick the landing were assessed through gymnastics‐specific performance criteria. Our results show that the expertise level significantly correlated with some performance variables. The more experienced the gymnasts, the better the hip and the knee angle, and the smaller the deviation from the desired vertical position. In addition, more experienced gymnasts had closed legs during the flight phase more often and took fewer steps after landing. Ultimately, they performed the jump with a higher quality in regard to the completion of the LA turn, which is not surprising as the LA turn is important for scoring in a competition. Overall, the results show an impact of the gymnast's expertise on performance. It would therefore be expedient to replicate our study with athletes who are the same age and have the same level of expertise to eliminate these two potential confounds as expertise and age are potentially related in this context (Munzert and Lorey 2013). A range of age and expertise levels was chosen for the study, as previous research has shown that imagery varies across different age groups (Dhouibi et al. 2021). When comparing different ages, Subirats et al. (2018) observed a transfer from a visual to a kinaesthetic imagery ability. Further, Liu et al. (2019) observed that the vividness of modalities and perspectives differs in individuals, which is reflected in a trend from a more internal to a more external imagery perspective over time. This is a finding, however, was not observable in the results of our study. Perhaps, the age difference was not large enough to have an impact. Another possibility is that the differences in imagery are more likely to be found in other sport‐specific analyses.

When comparing our findings with results from the existing literature, it should be noted that the present study only used three conditions (external, internal and neutral) whereas other investigations used more or different instruction conditions. For example, a standing long jump study (Marchant et al. 2018) and a cricket batting study (Bull et al. 2023) used two different external instructional conditions (proximal external, distal external) in addition to an internal and a control condition. A vertical jump study (Kons et al. 2021) used two internal instructional conditions (global internal, specific internal) in addition to an external condition. A dance study carried out by Gose and Abraham (2021) proposes a dynamic FoA. Further research could repeat the study with a sport‐specific imagery questionnaire and with adapted instructions, with more than two conditions.

Our results do neither directly support nor contradict the mechanisms of the Constrained Action Hypothesis (Wulf et al. 2001). Further, it seems to be less important where the attention is directed, but only that it is consciously directed. In our case, the Reinvestment Theory (R. Masters and Maxwell 2008) may apply, which assumes that performance can be disrupted when athletes attempt to consciously control movements. It states that when attention is consciously directed to execution‐relevant aspects of the movement sequence, automatic processes are disrupted (R. Masters and Maxwell 2008). This seems to apply to all three conditions, including the neutral condition, as attention is directed. All three instructions draw attention, either to the movement, to aspects of the execution or to intended movement effects. The imagery type does not seem to have any effect on it. Contrary to our hypothesis, matching the instruction‐guided FoA with the gymnast's most vivid imagery type, the performance did not appear more successful compared with the other conditions. The occurrence of different results could be explained by the different forms and intentions of the instructions (Werner and Federolf 2023). For example, the present study is only partially comparable with studies in which the goal is to perform a maximum vertical jump. In the study where the task was a maximum vertical jump with a 180° LA turn, the participants with an external FoA showed an improved jump height (Abdollahipour et al. 2015). As the instruction in the present study did not target maximum jump height, no improvement in jump height was expected and was not observed. In the present study, the FoA for the external and internal condition were designed to affect the LA turn. However, it does not seem to be as easy to target a certain movement part with externally focussed instruction as on the performance outcome or environment (Neumann 2019).

### 4.1 Limitations

The present study used the VMIQ‐2 as did other investigations before (e.g., Callow et al. 2013). The literature suggests that imagery modalities and perspectives can influence skill development and performance in several ways (Callow et al. 2013). In regard to the results of the present study should be noted that possible imagery preferences may differ between everyday movements and sport‐specific movements. Therefore, this non‐sport specific measure of imagery vividness may not be transferable to skill‐specific imagery features. The athletes were very similar in their response behaviour regarding the imagery vividness of everyday movements. Possible differences would more likely be observable in an assessment of gymnastics‐specific movements. However, a validated, context‐specific assessment tool were unavailable and therefore the one used was the most suitable for us.

Some limitations of the study need to be considered when formulating instructions to achieve a certain focus. The decline observed with the manipulation check indicates that the FoA have not been achieved as expected and was not sufficiently pronounced or sustained. It can be assumed that the retention of the FoA decreased continuously over the four rounds, as the values of the manipulation check decreased. Furthermore, the result may have been influenced by the exclusion of trials with insufficient main check values from the statistics. For the analysis, the limitation must be mentioned that trials with too much deviation from the desired LA turn were excluded because the camera position did not allow a correct analysis. This meant that the worst trials of the performance variable *Body2* were not taken into account for the analysis. Perhaps, differences would have appeared if the excluded trials had been taken into account. Both the excluded trials due to manipulation checks and due to insufficient variable Body2 were from different participants belonging to different groups. Most of them were from the no‐match group, which was also the largest group. However, all the excluded trials were predominantly from phases 4 and 5.

### 4.2 Conclusion

In order to utilise the positive effects of instruction‐guided attentional focus, the aim of the instruction should be taken into account (see Werner and Federolf 2023). It is necessary to make a distinction as to whether the instruction‐guided attentional focus is part of the motor learning process, serves technique correction or stabilises the movement (see Lawrence et al. 2011; Winkelman et al. 2017). Werner and Federolf (2023) suggest to consider the specific wording of the instruction to initiate the required FoA. In any case, the instruction should include a movement task. According to Raisbeck and Yamada (2019) it is necessary that the instruction addresses the movement goal to the detail of the movement an athlete aims to improve. The results suggest that focusing on one or the same aspect is not expedient for such a complex and mastered movement as is the case in the present study. It is not so relevant whether external, internal, or neutral, also because a distinction is not entirely clear depending on the wording. Also in order to make the high number of trials of the same movement more effective, the instruction‐guided FoA should differ. A dynamic form of instructions could be suitable here, which could counteract the focus on just one aspect (Gose and Abraham 2021) and, among other things, prevent a drop in concentration.

Subsequent studies should use a sport‐specific questionnaire to test imagery, as possible preferences could be different from those for everyday movements. Further studies should also adapt the instructions in order to place the external FoA on a more distant target as well as integrating an instruction that does not guide the FoA. In order to draw conclusions from the research for use in practice, it is recommended that especially evidence from the same sport and similar skills are taken into account. It is also important to consider the wording of the instruction and the movement goals addressed.

## Funding

The authors have nothing to report.

## Ethics Statement

This research was conducted in accordance with the Declaration of Helsinki and was approved by the Ethics Committee of the German Sport University Cologne, No. 138/2021.

## Consent

All participants, and their guardians if they were under 18 years of age, signed an informed consent form before the start of the experiment.

## Conflicts of Interest

The authors declare no conflicts of interest.

## Data Availability

The data that support the findings of this study are available from the corresponding author upon reasonable request.
